# Pulmonary benign metastasizing leiomyoma: report of three cases

**DOI:** 10.1186/1477-7819-11-281

**Published:** 2013-10-20

**Authors:** Hyun Woo Jeon, Soo Hwan Choi, Sook Whan Sung, Jae Kil Park

**Affiliations:** 1 Department of Thoracic and Cardiovascular surgery, Bucheon St. Mary’s Hospital, South Korea, Republic of Korea; 2 Department of Thoracic and Cardiovascular surgery, Seoul St. Mary’s Hospital, The Catholic University of Korea, Seoul Seo Cho Gu Ban Po Dong Banpo road 222, 137-701, South Korea, Republic of Korea

**Keywords:** Benign metastasizing leiomyoma, Uterine leiomyoma, Wedge resection

## Abstract

Benign metastasizing leiomyoma is very rare and usually occurs in women who undergo hysterectomy and myomectomy for uterine leiomyoma. This is a benign spindle-shaped smooth muscle cell tumor pathologically but metastasizes to the extrauterine organs. Lungs are the most common site of metastasis. We observed three cases of pulmonary benign metastasizing leiomyoma.

## Background

Uterine leiomyoma is the most common benign tumor in gynecology. It is a smooth muscle tumor with spindle cells. Very rarely, it spreads to the extrauterine organs as benign metastasizing leiomyoma. It is benign pathologically but malignant clinically. Lungs are the most common site of metastasis. There is controversy with the pathogenesis and treatment modality of this condition. We present three cases of benign metastasizing leiomyoma.

## Case presentation

### Case 1

**Figure 1:**
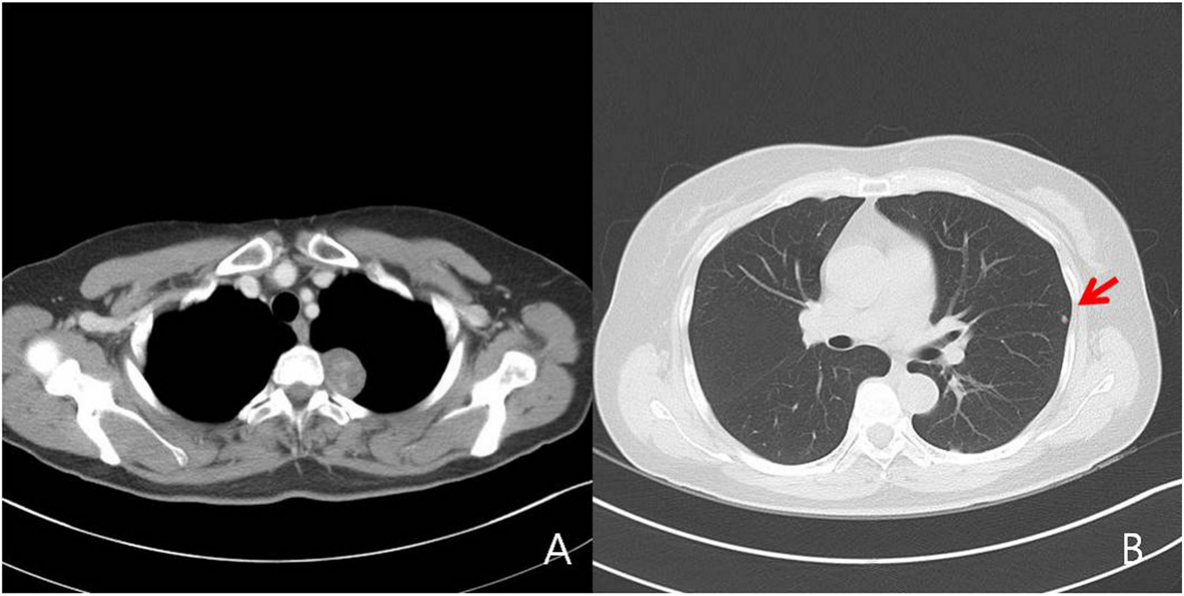
Well-defined mediastinal mass was located at the paravetebral space (A) and tiny nodule was involved in LUL (arrow) (B).

A 53-years-old woman was referred for right ovarian mass. She complained of urinary frequency for several months. In 2008, uterine myoma was diagnosed. Pelvic magnetic resonance imaging (MRI) showed a 10.7-cm cystic mass with solid portion in right adnexa. Chest X-ray showed an ovoid mass (2.5 × 3.1 cm) at left paravertebral space. Chest computed tomography (CT) revealed a well-defined oval mass at left paravertebral space and tiny nodule involving left upper lobe (Figure 1). CA125 and CA 19–9 were 34.97 U/mL and 17.04 U/mL, respectively. After total hysterectomy with bilateral salpingo-oophorectomy, mediastinal mass excision and pulmonary wedge resection were performed by video-assisted thoracic surgery (VATS). Histopathology of pelvic mass showed endometroid adenocarcinoma with uterine leiomyoma. Microscopic examination of pulmonary lesion showed well-encapsulated and proliferation of uniform spindle cells without mitosis and necrosis. Pleural mass was diagnosed as lymphangioma on pathologic examination. During 22 months of follow-up, there was no evidence of tumor recurrence.

### Case 2

**Figure 2:**
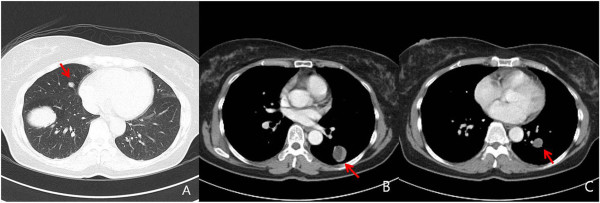
**Total of five well-defined masses located in both lungs (A,B,C).** The largest one is in RLL **(A)**.

**Figure 3:**
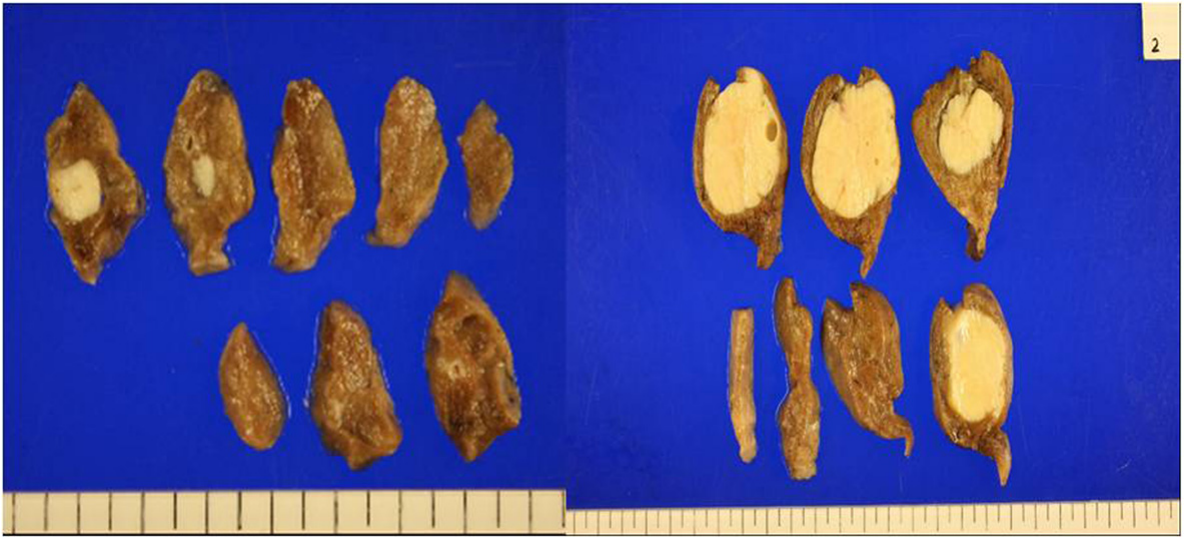
On the whole, these tumors were well-marginated, ovoid, and firm.

A 57-year-old woman was referred for abnormal chest X-ray. She was asymptomatic with normal physical examination and lab findings. She had undergone total hysterectomy for uterine leiomyoma 22 years before. Chest CT showed a total of five masses in both lungs (Figure 2). The masses were oval and well-marginated. The maximal size was 5.3 × 3.8 cm involving the right lower lobe (RLL). These lesions showed mild activity on positron emission tomography (PET). Percutaneous needle biopsy was performed. Histology revealed benign metastasizing leiomyoma. Wedge resection was performed through bilateral minithoracotomy. Peripheral masses showed visceral protrusion without retraction. The masses were well-defined, firm, and oval-shaped (Figure 3). Histopathology revealed proliferation of smooth muscle cells without mitosis, pleomorphism, and necrosis. The tumors were positive for smooth muscle antigen (SMA), estrogen, and progesterone receptors. During 20 months of follow-up, there was no evidence of tumor recurrence.

### Case 3

**Figure 4:**
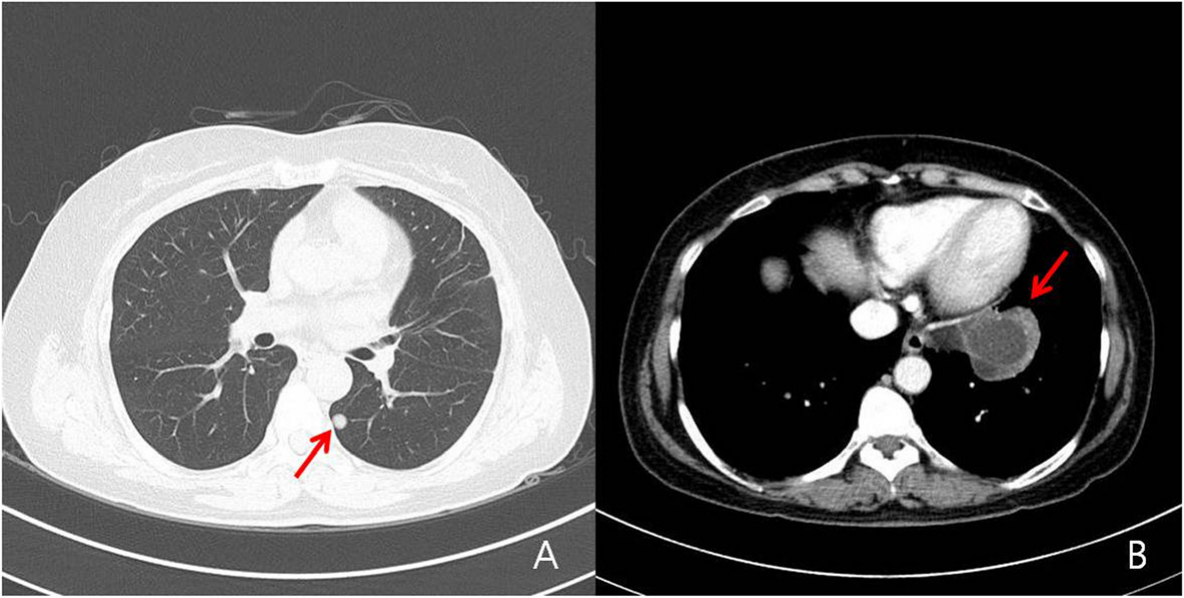
Chest CT showed cystic lesion involving LUL (arrow) (B) and well-defined mass involving LLL (arrow) (A).

**Figure 5:**
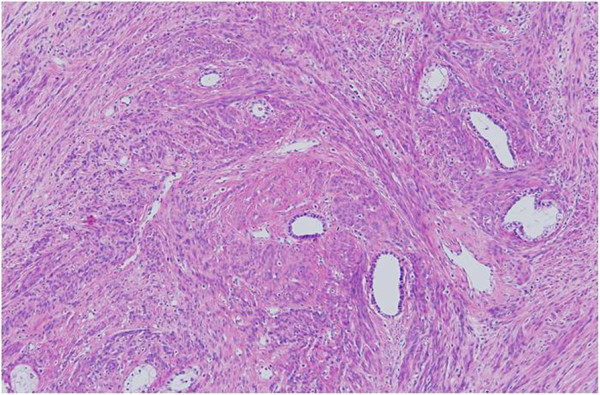
**There are fascicles of smooth muscle cells and entraped bronchiolar structure.** H&E stain (×200).

A 56-year-old woman was referred for abnormal chest X-ray. She had undergone total hysterectomy for uterine leiomyoma 30 years before. Chest CT showed two cystic lesions in left upper lobe (LUL) (5.5 × 4.5 cm) and tiny nodule in left lower lobe (LLL) (Figure 4). Clinical impression was congenital cystic adenomatoid malformation (CCAM). After 1 year of follow-up, the lesions did not increase in size. Wedge resection for LUL and LLL was performed through VATS. White-colored solid masses were found. Histopathology revealed proliferation of smooth muscle cells without mitosis, pleomorphism, and necrosis (Figure 5). During the 1-year follow-up period, there was no evidence of tumor recurrence.

## Discussion

Benign metastasizing leiomyoma is a very rare disease. Steiner was the first to report this condition in 1939 [1]. The definition of benign metastasizing leiomyoma is slow-growing extrauterine smooth muscle tumors with a history of surgery for uterine leiomyoma or presence of uterine leiomyoma. It is a benign condition. Lungs are the most common sites of metastasis. Most cases were reported in women of reproductive age. Disease-free interval was 3 months to 20 years after hysterectomy [2]. Patients are usually asymptomatic and the lesions are identified on imaging study. Most cases showed multiple metastases and single metastasis is very rare [3]. Clinical course is usually indolent [4] as leiomyoma is less than five mitoses per 10 HPFs without cellular atypia and necrosis. Well-defined, oval-shaped nodules ranging in size from a few millimeters to several centimeters are typical radiographic findings [2]. Infrequently, cystic lesions [5], cavitary formation, and military patterns are also reported.

Surgical resection is the widely accepted option for the resectable tumors [6]. These lesions are affected by sex hormones [4]. In inoperable cases, bilateral oophorectomy and hormonal therapy may be alternative approach.

## Conclusions

In conclusion, though benign metastasizing leiomyoma is rare, thoracic surgeons should consider this diagnosis in patients with a history of uterine leiomyoma. Resection should be considered for the resectable tumors.

## Consent

Written informed consent was obtained from the patient for publication of this case report with images.

## Competing interests

The authors declare that they have no competing interests.

## Authors’ constributions

HWJ is a first author and carried out review of medical record and drafted the manuscript. SHC carried out review of medical record and histology. SWS is a corresponding author and carried out revision of the manuscript. JKP carried out revision of the manuscript. All authors read and approved the final manuscript.
